# Transcriptome analysis of ciliary-dependent MCH signaling in differentiating 3T3-L1 pre-adipocytes

**DOI:** 10.1038/s41598-021-84138-4

**Published:** 2021-03-01

**Authors:** Laurie B. Cook, Henry D. Ophardt, Rongkun Shen, Bryan H. Pratt, Lucas A. Galbier

**Affiliations:** Department of Biology, 217 Lennon Hall, SUNY Brockport, 350 New Campus Drive, Brockport, NY 14420 USA

**Keywords:** Gene expression analysis, Cell signalling, Bioinformatics, Peptide hormones, Ciliogenesis, Differentiation, Obesity

## Abstract

An understanding of adipocyte responsiveness to G-protein-coupled receptor-(GPCR) derived signals must take into consideration the role of membrane microenvironments; that individual sub-populations of proteins may vary significantly across different regions of the cell, and that cell differentiation alters those microenvironments. 3T3-L1 pre-adipocytes undergo a dramatic phenotypic transformation during differentiation into adipocytes, requiring the development of a transient primary cilium. We demonstrate that melanin-concentrating hormone (MCH) receptor 1, a GPCR that stimulates appetite, translocates to the transient primary cilium during early 3T3-L1 cell adipogenesis. Furthermore, we used RNA-Seq to investigate whether MCH signaling is influenced by its receptor localization and whether MCH can influence the transcriptome of early adipocyte development. We found that MCH signaling is sensitive to receptor localization to cilia, and this alters the adipogenic transcriptional program. Also, novel MCH signaling pathways in 3T3-L1 cells are identified, including those for circadian rhythm, the inflammatory response, and ciliary biogenesis. The presence of active MCH-signaling pathways in pre-adipocytes and the discovery that these pathways intersect with the early adipogenic program, among other newly-identified signaling pathways, suggests that the use of MCH receptor 1 antagonists for clinical interventions may have unintended consequences on adipose tissue development.

## Introduction

One of the most common model systems for studying fundamental adipocyte mechanisms is the 3T3-L1 pre-adipocyte. Originally derived from 17 to 19 day Swiss 3T3 mouse embryos^1^, these cells function like a fate-determined stem cell, differentiating into adipocytes over a ten-day treatment schedule initiated with insulin, dexamethasone, and isobutylmethylxanthene. These factors, coupled with growth-arrest and mitotic expansion, lead to the activation of key adipogenic transcription factors such as C/EBP-α, C/EBP-β, and PPAR-γ, which change the gene expression profile in these cells towards differentiation and the accumulation of lipid droplets^2^. Early in the differentiation process, Day 2 differentiating pre-adipocytes express a transient primary cilium that protrudes from the cell as an extension of the plasma membrane. This short-lived signaling structure may help to inhibit Wnt signaling, enabling adipogenesis to occur^3,4^. The primary cilium is therefore necessary for adipocyte development, since cells fail to fully differentiate in pre-adipocyte knock-out studies targeting the ciliary genes *ift88* and *kif3a*^5^.

The transient localization of receptors to the primary cilium is of particular interest because it can be highly regulated and defective receptor localization can cause disease^6^. Among the most characterized mutations are those involving parts of the BBSome complex, named for Bardett-Biedl Syndrome^7^. Researchers have traced a connection between childhood- and adult-onset obesity, a hallmark of BBS, to single nucleotide polymorphisms in several BBS genes^8^. Melanin-concentrating hormone (MCH) receptor 1 is a G protein-coupled receptor that depends on *bbs2* and *bbs4* for trafficking to the ciliary compartment in primary neurons, and ablation of these genes prevents MCH receptor 1 localization and signaling, resulting in the onset of BBS phenotypes^9^. The presence of MCH receptors in adipose tissue led researchers to investigate whether MCH alters adipose cell development or function. MCH action on rat adipocytes was shown to enhance synthesis and secretion of leptin^10^, and likewise, leptin was shown to feedback centrally to turn off synthesis and secretion of MCH from the brain^11^. We reported that MCH also facilitates the migration of pre-adipocytes through MCH receptor 1^12^, and this was supported by evidence that MCH stimulates monocyte migration as well^13^.

MCH may influence the differentiation or proliferation of adipose cells. Since MCH receptor 1 knock-out mice are resistant to diet-induced obesity, it seems likely that MCH promotes the development of adipose tissue^14^. Indeed, in support of this, Mul et al. discovered that centrally acting MCH enhances sympathetic adipose drive. Although this diminished the perceived role of peripheral MCH in controlling adipose tissue mass, they also reported that direct action of MCH on white adipose tissue influenced cell size, but not number, in a small in vivo rat study^15^. One possibility is that MCH may work to enhance lipid storage in adipocytes.

MCH receptor 1 has a ciliary-targeting sequence^9^ but the exact role of its localization to primary cilia is unknown. With the loss of MCH receptor 1 localization to primary cilia in the brain associated with Bardet-Biedl Syndrome^16^, and new data shown here placing MCH receptor 1 in the primary cilia of differentiating adipocytes, we decided to further explore the impact of ciliary-localized MCH receptor 1 on MCH signaling using transcriptome analysis (mRNA sequencing). By quantifying MCH-mediated changes in gene expression in ciliated and non-ciliated cells, we demonstrate the importance of membrane microenvironment in controlling GPCR signaling, and we provide convincing evidence that MCH signaling in developing pre-adipocytes acts to inhibit the adipogenic commitment.

## Results and discussion

### MCH receptor 1 is localized to primary cilia in differentiating 3T3-L1 cells

**Figure 1:**
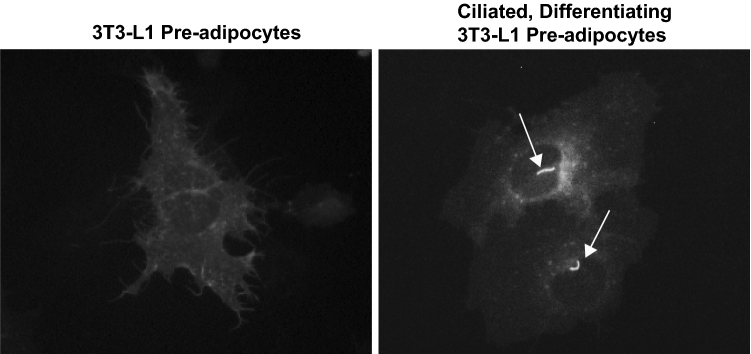
MCH receptor 1 localizes to different membrane structures in 3T3-L1 preadipocytes. 3T3-L1 preadipocytes were transiently transfected with MCHR1-eYFP in 35 mm dishes on coverslips. Some dishes were initiated to differentiate for 2 Days (right panel). All cells were fixed with 4% paraformaldehyde. MCHR1-positive putative cilia are indicated with arrows (right panel).

We hypothesized that MCH receptor 1 would be localized to the plasma membrane of 3T3-L1 cells, but also primary cilia when they appeared on Day 2 of our 10-day differentiation protocol. The experiment in Fig. 1 shows the cellular localization of transiently-transfected eYFP-tagged MCHR1 expressed in 3T3-L1 pre-adipocytes. The localization of MCH receptor 1 depended upon the developmental stage of the cells, moving from the cell surface of pre-adipocytes (Fig. 1, left panel), to a tiny cellular appendage indicated by the arrows (Fig. 1, right panel) during early differentiation.

**Figure 2:**
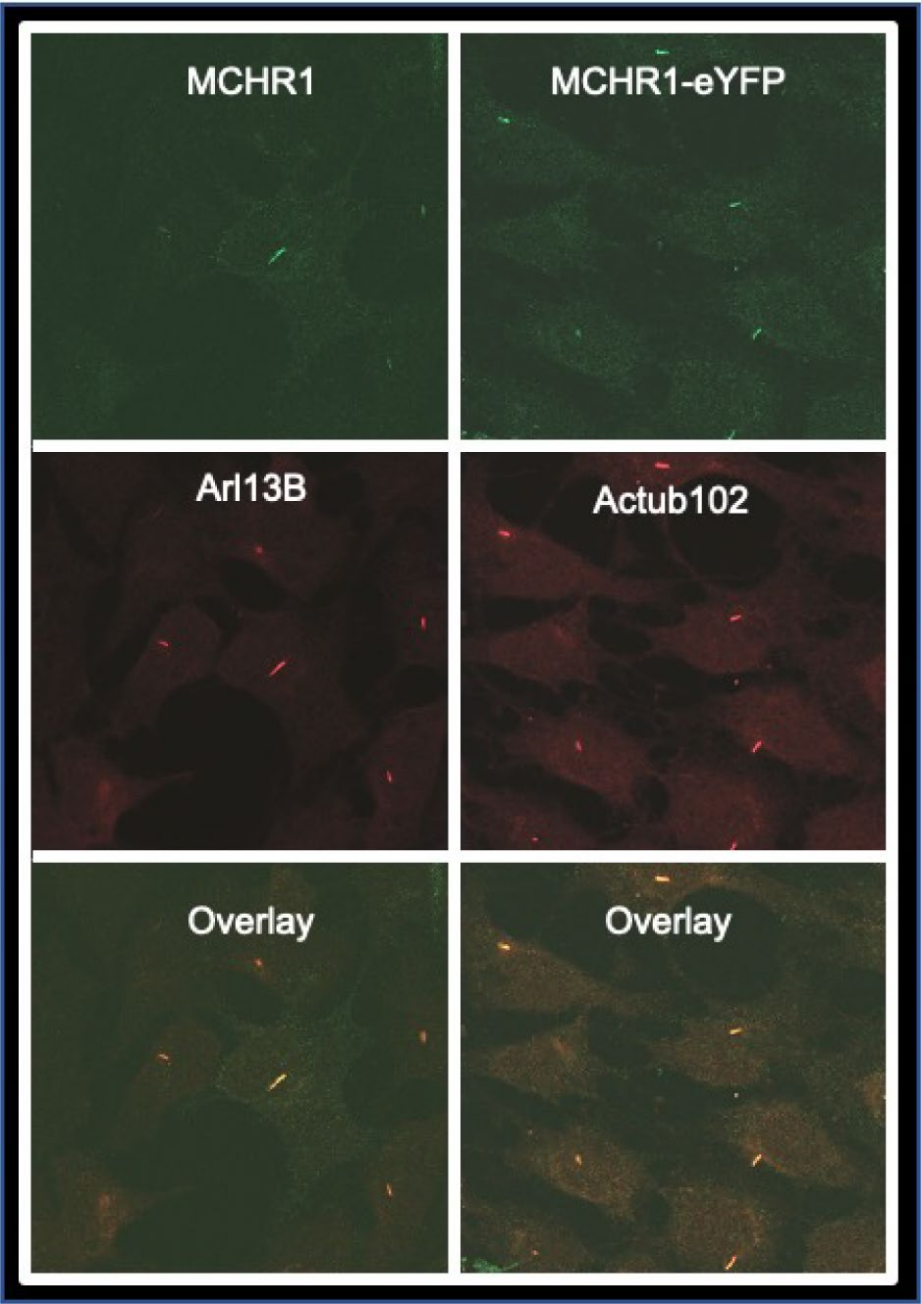
Co-localization of MCH receptor 1 (MCHR1) with ciliary markers. 3T3-L1 cell adipogenesis was initiated with differentiation media for 2 days. *Left panels:* Endogenous MCHR1 was detected using anti-MCHR1 antibody. Arl13B-antibody was used to label primary cilia. *Right panels:* MCHR1-eYFP was transiently transfected into cells prior to differentiation treatment. Anti-Actub102 was used as a ciliary marker. The Overlay panels illustrate colocalization.

To better determine if MCHR1 is actually localizing to primary cilia of 3T3-L1 cells on Day 2, we performed confocal microscopy on cells immunostained with primary antibody to MCH receptor 1 (Fig. 2, left panels) or cells expressing MCH receptor 1 tagged with eYFP (Fig. 2, right panels) plus antibody towards two primary cilia markers, acetylated tubulin and ARL13B. The overlap between MCH receptor 1 and the ciliary marker patterning indicates that the receptor is indeed localized to the primary cilia in differentiating 3T3-L1 cells. MCH receptor 1 has been previously localized to the primary cilia in neurons^16^, but the significance of this remarkable receptor translocation is unknown. Primary cilia of neurons are quite stable^17^, whereas that of the developing 3T3-L1 preadipocyte are transient, lasting less than 48 hours^5^. Our model system provided a unique environment in which to study the role of primary cilia in regulating MCH receptor 1 signaling. Our samples, taken from non-ciliated 3T3-L1 cells, and Day 2 differentiating and ciliated 3T3-L1 cells provided a tool to capture transcriptomes under different conditions to determine whether MCH signaling is changed when its receptor is localized to primary cilia. It also gave us an opportunity to determine whether MCH could alter the progression of early adipogenesis.

### Transcriptome analysis reveals MCH-mediated changes in gene expression that are influenced by primary cilia

Gene expression changes were studied to determine the following: (1) whether the cellular differentiation program toward adipocyte formation was activated, (2) whether MCH treatment influenced this cellular differentiation, and (3) whether primary cilia formation affected MCH signaling. Cells were treated with or without 100 nM MCH for 6 h prior to total RNA extraction. Triplicate RNA-Seq samples were used in the experimental design to minimize the effect of sample variation and increase statistical robustness.

Following RNA sequencing, a large dataset containing sequencing reads was produced from each of the twelve samples. The beginning and tailing part of the 150 bp read were generally having poor quality; these were trimmed, which then focused on bases from 7 to 106. Reads were aligned against the mouse mm10 genome assembly and RefSeq annotation. DeSeq2, an R/Bioconductor package, was used to identify statistically significant changes in transcript levels between the four conditions in pairwise manner: ciliated vs non-ciliated, plus vs minus MCH. Four comparison results were produced that analyzed the transcriptional profiles of: ciliated cells; non-ciliated cells; ciliated cells treated with MCH; and non-ciliated cells treated with MCH.

**Figure 3:**
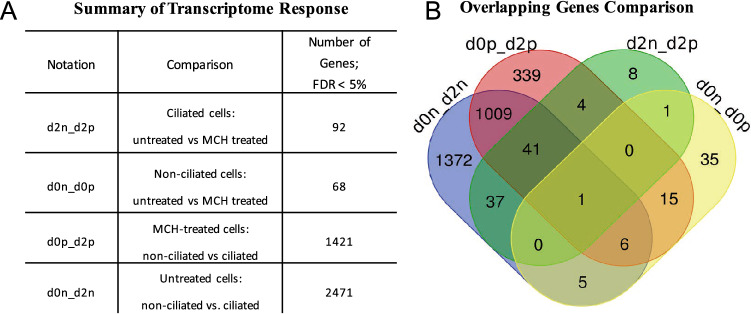
RNA-Seq was performed on total RNA samples isolated from either 3T3-L1 pre-adipocytes or Day 2 differentiating pre-adipocytes harboring primary cilia. Some samples were pretreated with 100 nM MCH prior to harvesting. (**A**) The number of responding genes reaching statistical significance with a False discovery rate (FDR) of < 0.05 is shown. (**B**) A Venn Diagram illustrating overlapping genes for all comparison groups.

**Figure 4:**
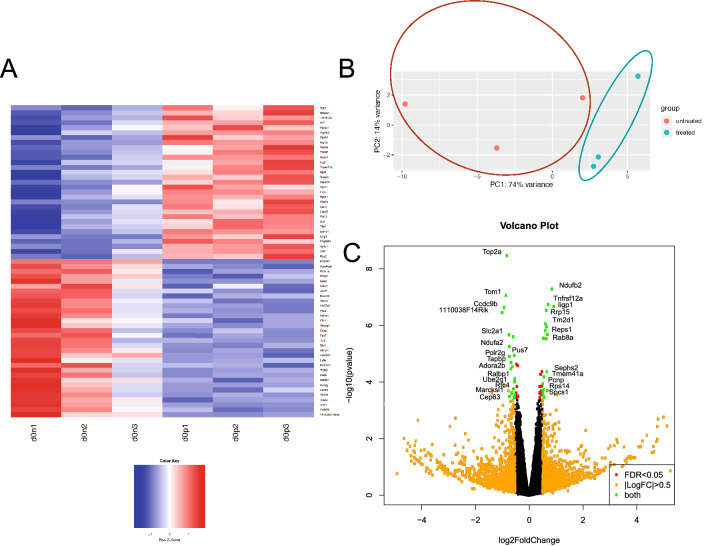
Differentially expressed genes in non-ciliated 3T3-L1 cells treated with 100 nM MCH. (**A**) A heatmap illustrating agreement between triplicate RNA-Seq datasets comparing expression changes between MCH treated (d0p1,2,3) untreated (d0n1,2,3) conditions with *p* < 0.05. Each row illustrates a gene. Red colors indicate increased gene expression in MCH-treated samples. Blue indicates decreased gene expression in MCH-treated samples. (**B**) Principal component analysis (PCA) was used to show that samples cluster into two major groups, where the first two principal components were plotted (PC1 and PC2). (**C**) Volcano plot illustrates those genes that passed prefiltering criteria. Genes with |LogFC:Fold Change|> 0.5 are shown in orange; genes with FDR < 0.05 are shown in red; with those in green passing both criteria; some of which are labeled.

Figure 3 reflects the number of genes that are transcriptionally altered between the conditions stated. False discovery rate (FDR) was used to determine statistical significance; data for FDR < 5% are shown. The number of genes responsive to MCH in 3T3-L1 cells is very small with only 68 genes changing expression in unciliated cells and 92 genes in ciliated cells. Interestingly, there are only 2 genes overlapping between those 2 lists. When the triplicate data set was explored in more detail, we found good agreement across replicates (Fig. 4A) and clear differentiation between clusters using a principal component analysis (Fig. 4B). Select genes passing statistical filtering criteria are shown in Fig. 4C.

**Figure 5:**
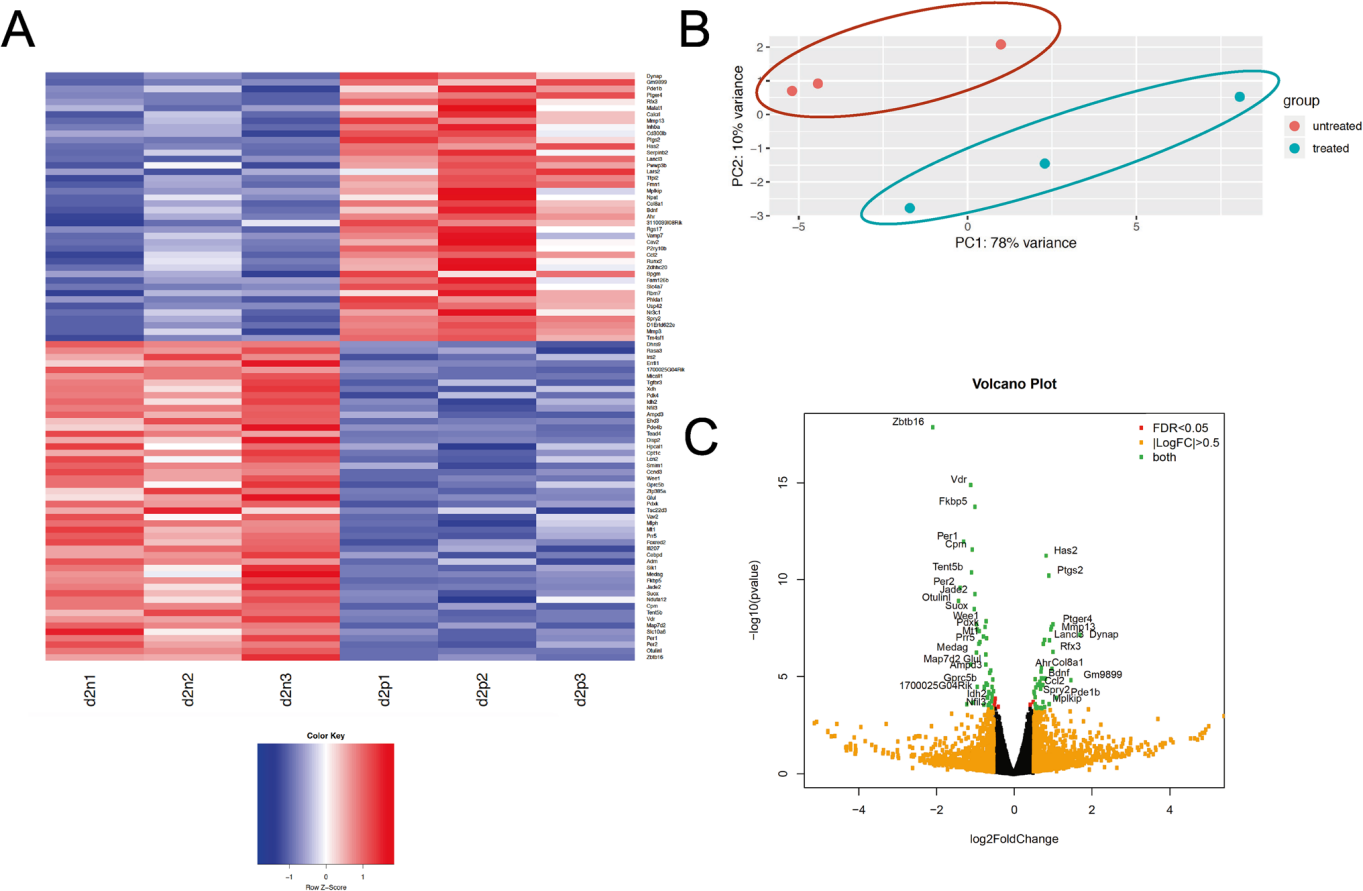
Differentially expressed genes in ciliated 3T3-L1 cells treated with 100 nM MCH. (**A**) A heatmap illustrating agreement between triplicate RNA-Seq datasets comparing expression changes between MCH treated (d2p1,2,3) untreated (d2n1,2,3) conditions with *p* < 0.05. Each row illustrates a gene. Red colors indicate increased gene expression in MCH-treated samples. Blue indicates decreased gene expression in MCH-treated samples. (**B**) Principal component analysis (PC) was used to show that samples cluster into two major groups, where the first two principal components were plotted (PC1 and PC2). (**C**) Volcano plot illustrates those genes that passed prefiltering criteria. Genes with |LogFC|> 0.5 are shown in orange; genes with FDR < 0.05 are shown in red; with those in green passing both criteria; some of which are labeled.

It seems that the initiation of the 3T3-L1 cell differentiation program, highlighted by the appearance of a primary cilium, causes activation of a gene expression program elucidating changes in over 2,400 genes (FDR < 5%) in just two days, but when cells are treated with MCH, only about 1,400 genes (FDR < 5%) are differentially expressed as shown in Fig. 3, suggesting that MCH signaling alters the transcriptional program that drives differentiation of preadipocytes. Comparisons of triplicate agreement of ciliated 3T3-L1 cells treated with and without MCH is shown via Fig. 5. There is clearly a cluster of genes on Day 0 and Day 2 that respond to MCH, some genes increasing expression and some genes decreasing expression according to the principal component analysis. This data indicates that MCH signaling alters the transcriptome in both ciliated and non-ciliated 3T3-L1 cells. Although genes for hypoxanthine–guanine phosphoribosyltransferase (*hprt),* RNA pol II and RNA Pol III were validated as acceptable reference genes for 3T3-L1 cell development^18^, only RNA Pol II was used for comparative calculations throughout this study.

### Validation of RNA-Seq dataset using known regulators of adipogenesis

**Figure 6:**
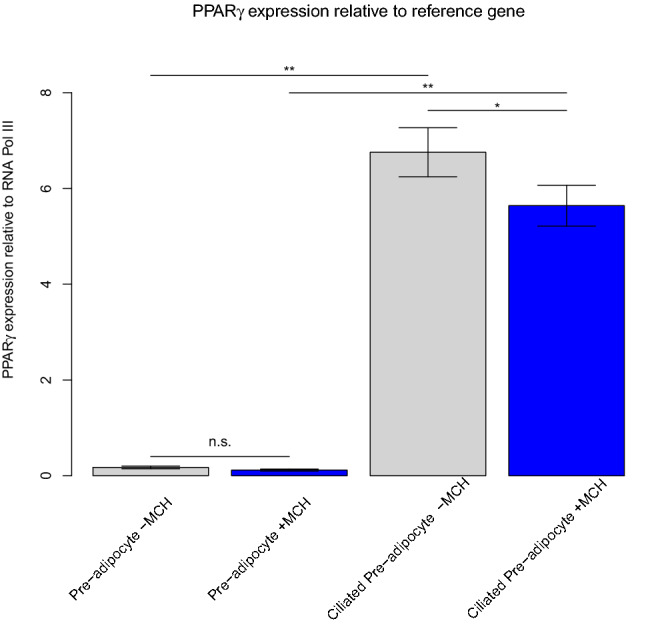
Expression of *ppar-γ* compared to reference gene, analyzed by qPCR. Expression is significantly increased in differentiating ciliated 3T3-L1 cells versus non-ciliated 3T3-L1 cells. Furthermore, 6 h 100 nM MCH treatment results in decreased expression at both timepoints of differentiation (n = 3). *p < 0.05; **p < 0.01; ***p < 0.001; n.s. not significant.

The combination of growth arrest with the activation of key adipogenic transcription factors such as *c/ebp-α*, *c/ebp-β*, and *ppar-γ,* change the gene expression profile in these pre-adipocytes resulting in the accumulation of lipid droplets and conversion to an adipocyte^19^. Thus, it would seem like these key genes would be best to validate our dataset. However, elevated *c/ebp-β, c/ebp-δ* and *ppar-δ* gene expression occurs just prior to *ppar-γ* induction, and Gregoire et al. suggest that it is the low levels of these genes that keeps the pre-adipocyte from fully committing to differentiation^20^. It wasn’t surprising that these genes didn’t stand out as greatly upregulated in ciliated pre-adipocytes when compared to non-ciliated pre-adipocytes during the qPCR validation process as shown in Table 1. However, *ppar-γ* was found to be highly upregulated (> fivefold) using the qPCR method as opposed to only slightly upregulated (1.3-fold) using RNA-Seq (Fig. 6). We propose that differing sensitivities in the sequence amplifications may be contributing to the varied outcomes. Importantly though, both methods agree that the gene was upregulated in ciliated cells when compared to non-ciliated cells, and the sheer number of significantly changed genes in ciliated 3T3-L1 cells compared to unciliated 3T3-L1 cells supports the claim that the differentiation program has been successfully initiated.

**Table 1:** RNA-Seq results show slight upregulation of master regulatory genes for adipogenesis when comparing non-ciliated and ciliated 3T3-L1 samples.

Gene	Fold change	p-value
*ppar-δ*	1.52	6.90E−03
*ppar-γ*	1.30	3.30E−02
*c/ebp-α*	1.74	2.78E−02
*c/ebp-β*	n.s	4.18E−01
*c/ebp-δ*	1.68	8.53E−06

*n.s*. Not significant.

Regulation of the adipogenic program by MCH receptor 1 is of great interest. Despite Mul et al.*’s* findings that sympathetic drive is largely responsible for MCH-mediated effects on adipose tissue, their findings that direct action of MCH on adipose tissue influences cell size, but not the number of cells, leaves open the possibility that circulating MCH agonists or antagonists could positively affect human health^15^. Recently, Hilgendorf and colleagues made a case for ciliary-localized omega-3 fatty acid signaling through FFAR4 coupling to G_s_ as a primary control mechanism for the preadipocyte to adipocyte switch in vivo^21^. 3T3-L1 cells are also known to be responsive to MCH, and the literature includes reference to MCH mediating both G_i_- and G_q_-mediated responses^22^. We next selected three of the most significantly expressed genes for further validation of early adipogenesis (non-ciliated compared to ciliated pre-adipocyte) by qPCR. The gene for lipocalin 2, *lcn2*, codes for an adipokine which has been previously shown to be rapidly upregulated during 3T3-L1 pre-adipocyte differentiation^23^. We confirm this dramatic upregulation in *lcn2* expression in ciliated cells in both our RNA-Seq (69-fold) and qPCR (76-fold) experiments. Interestingly, MCH caused a 0.62-fold decrease (*p* < 0.05) in *lcn2* expression as measured by RNA-Seq, suggesting that MCH receptor 1 signaling in primary cilia partially inhibits the adipogenesis program; this is supported by a slight, but statistically significant decrease in *ppar-γ* expression due to MCH treatment (Fig. 6).

*Igfbp4* has been identified as a key regulator of adipogenesis and is also appropriately upregulated in our ciliated pre-adipocytes (6.9-fold via RNA-Seq and 3.7-fold via qPCR), indicating that our adipogenic induction is strong^24^. Growth arrest-specific gene 7 (*gas7*) regulates mesenchymal stem cell differentiation into osteoblasts and is upregulated when differentiation is induced with dexamethasone^25^. Since osteoblasts and adipocytes have a shared lineage and transdifferentiation has been reported^26^, it isn’t too surprising that we also see upregulation of this gene early during 3T3-L1 cell differentiation (6.2-fold via RNASeq and 1.1-fold via qPCR). In conclusion, we are confident that early adipogenesis was initiated in our Day 2 samples. Our data indicates that, at least in early adipogenesis, that MCH signaling in cilia prevents the adipogenic switch from fully activating, and it is highly likely that G_i_ signaling is in part responsible, since G_s_ signaling to cAMP is a necessary step in early adipogenesis^27^.

### MCH-mediated transcriptional changes in non-ciliated 3T3-L1 preadipocytes

We initially used a PANTHER Pathway Overrepresentation Test (PANTHER version 14.1; Overrepresentation Test Released 03/12/2019) to determine if certain pathways were statistically overrepresented in our non-ciliated 3T3-L1 cell data set comparing untreated to MCH-treated pre-adipocytes^28^. The *Mus musculus* reference list was used with a Fisher’s Exact Test with FDR multiple test correction. When only results with FDR *p* < 0.05 were considered, there were no statistically significant hits. We screened the same data using the Reactome Bioinformatics Tool against their human homologues, focusing on hits with *p*-values < 0.05^29^ and obtained a more extensive list of pathways in undifferentiated pre-adipocytes affected by MCH (Supplementary Table 1). Of particular interest to this study are the four clusters of interleukin signaling pathway (Interleukin-4, -13, -10, and -12) genes, suggesting MCH may be influencing the inflammatory response, and the “cilium assembly” pathway genes (4 genes; *cep63, tuba1c, rab8a, hsp90aa13; p*-value 3.98E−03), suggesting MCH may be influencing early ciliogenesis.

Interleukin-4 (IL-4) and Interleukin-13 (IL-13) expression in 3T3-L1 cells is correlated with lowered lipid storage, while IL-4 has specifically been shown to inhibit adipogenesis and activate lipolysis by stimulating HSL activity. If 3T3-L1 cells can influence the migration of eosinophils then this crosstalk between adipocytes and eosinophils may contribute to chronic inflammation in adipose tissue and adipocyte maturation^30^. We demonstrate that MCH can influence the production of cytokines by differentiating adipocytes. These data, together with reports that MCH knockout-mice have lowered levels of macrophages infiltrating intestinal tissues^13^, strongly support a role for MCH in mediating the inflammatory condition.

Hamamoto et al. have reported that in hRPE1 cells transfected with MCH receptor 1, MCH triggers primary cilia shortening through Akt, so MCH signaling has previously been coupled to ciliary stability^31^. This study, however, is the first time MCH has been implicated in de novo ciliary assembly and this has implications for its function in the central nervous system as well. Not only was altered ciliary assembly gene expression identified as a cluster in non-ciliated cells treated with MCH, but interestingly, the gene for M-Phase Specific PLK1 Interacting Protein (*mplkip*) was also upregulated 1.68 fold (*p* < 0.05) with MCH in ciliated cells, and its centrosomal location and putative interaction with Plk1 suggest a role for MCH signaling in regulating centriolar duplication and/or ciliary biogenesis^32^.

### MCH-mediated transcriptional changes in ciliated 3T3-L1 preadipocytes

DAVID^19^, was again used as a starting point to cluster the 92 differentially expressed genes responsive to MCH, this time in ciliated 3T3-L1 preadipocytes, and this resulted in the emergence of a few interesting patterns. Eleven genes clustered into the most significant “regulation of cell proliferation” category (*adm, bdnf, calcrl, cav2, ccl2, irs2, ptgs2, runx2, spry2, tgfbr3 & zbtb16; p*-value 1.9E−04) with six of these (*bdnf, cav2, ptgs2, spry2, tgfbr3 & zbtb16*; *p*-value 4.0E−03) in the “negative regulation of cell proliferation” category. Other significant cluster categories include “phosphoproteins” (40 genes; *p*-value 3.0E−03) and “metal binding” (21 genes; *p*-value 7.3E−03).

Interestingly, when 50 down-regulated genes responding to MCH treatment of ciliated preadipocytes were subjected to PANTHER Pathway Analysis, Purine Metabolism (2 genes, *ampd3* & *xdh; p*-value 1.54E−04; FDR 2.53E−02) and Circadian Clock System (2 genes, *per1* & *per2; p*-value 2.35E−04; FDR 1.93E−02) genes were found to be moderately overrepresented. A follow-up GO-Slim Biological Process Analysis also re-identified *per1* and *per2,* as well as a third Circadian Rhythm gene, *nfil3* (79.06-fold enrichment; *p*-value 1.13E−05; FDR 2.02E−02). We again screened the same data using the Reactome Bioinformatics Tool against their human homologues, focusing on hits with a FDR < 0.05^29^. These genes plus a fourth gene, *sik1*, appeared in the “circadian rhythm” pathway (*p*-value 3.54E−07). Period 2 (*per2*) has been shown to inhibit adipogenesis through its interactions with PPARγ^33^ and Period 3 (*per3*) prevents development of adipocytes from mesenchymal stem cells^34^. *Nfil3*, the third clock gene identified as a gene sensitive to MCH, was previously identified as a linker between the microbiota, circadian clock proteins and the host cells. Microbiota may act to increase *nfil3* expression in epithelial cells of the intestine, which in turn regulates lipid storage and fat deposition^35^.

When 42 up-regulated genes responding to MCH treatment of ciliated preadipocytes were also subjected to the same PANTHER Pathway Analysis, Plasminogen Activating Cascade (3 genes, *mmp3, mmp13 & serpinb2; p*-value 4.32E−06; FDR 7.09E−04) genes were > 100 fold enriched. A follow-up GO-Slim Biological Process Analysis identified Extracellular Structure Organization genes as well (4 genes, *has2, mmp3, mmp13 & col8a1; p*-value 7.13E−06; FDR 6.38E−03).

When we screened the full set of data for significantly changed genes with the Reactome Bioinformatics Tool (*p*-values < 0.05)^29^, we again obtained a more extensive list of pathways in ciliated pre-adipocytes affected by MCH (Table 2). Pathways related to interleukin signaling reappeared. It seems that MCH can also elicit mRNA expression changes in pathways governing nuclear receptor signaling. The “nuclear receptor transcription” pathway included 2 genes (*nr3c1* and *vdr, p*-value 6.37E−09) and the “signaling by nuclear receptors” pathway included 5 genes *(cav2, mmp3, pdk4, dhrs9, fkbp5*; *p*-value 2.28E−02). Additionally, a fifth gene involved in the “circadian clock” pathway (p-value 4.62E−06), *nr3c,* was identified. Our finding that the gene for the Vitamin D receptor was the most up-regulated gene in ciliated cells in response to MCH was at first surprising to us, but upon further examination and when placed in the context of the complete transcriptome response in ciliated adipocytes, it becomes clearer. Vitamin D regulates the inflammatory response in adipocytes^36^, and modulation of receptor availability by MCH could be a means by which the inflammatory response is controlled. Expression of *vdr* is elevated in early adipogenesis and it is hypothesized that 1,25(OH)2D3 could exert an anti-adipogenic effect via Wnt/b-catenin. In fact, bone marrow stromal cells are precursors to the osteoblast/adipocyte lineage and the Vitamin D receptor inhibits adipogenesis in these cells by suppressing inhibitors of the Wnt signaling pathway^37^.

**Table 2:** Reactome pathway analysis of genes changed by MCH in ciliated 3T3-L1 Cells.

Pathway identifier	Pathway name	p-value	Genes found in pathway
R-HSA-383280	Nuclear receptor transcription pathway	6.37E−09	*Nr3c1;Vdr*
R-HSA-6785807	Interleukin-4 and Interleukin-13 signaling	3.12E−06	*Mmp3;Ccl2;Cebpd;Lcn2;Ptgs2*
R-HSA-400253	Circadian Clock	4.62E−06	*Per2;Per1;Nr3c1;Nfil3;Sik1*
R-HSA-8941332	RUNX2 regulates genes involved in cell migration	8.39E−06	*Mmp13;Runx2*
R-HSA-1592389	Activation of matrix Metalloproteinases	1.68E−05	*Mmp13;Mmp3*
R-HSA-1442490	Collagen degradation	3.81E-05	*Mmp13;Mmp3;Col8a1*
R-HSA-2032785	YAP1- and WWTR1 (TAZ)-stimulated gene expression	5.80E-04	*Runx2;Tead4*
R-HSA-212436	Generic transcription pathway	1.66E-03	*Mmp13;Nr3c1;Ccnd3;Prr5;Zfp385a;Zbtb16;Bdnf;Vdr;Fkbp5;Runx2;Tead4*
R-HSA-8878166	Transcriptional regulation by RUNX2	2.04E−03	*Mmp13;Nr3c1;Runx2*
R-HSA-1474228	Degradation of the extracellular matrix	2.11E−03	*Mmp13;Mmp3;Col8a1*
R-HSA-449147	Signaling by interleukins	2.90E−03	*Vamp7;Mmp3;Rasa3;Ccl2;Irs2;Cebpd;Serpinb2;Lcn2;Ptgs2*
R-HSA-2022090	Assembly of collagen fibrils and other multimeric structures	3.08E−03	*Mmp13;Mmp3;Col8a1*
R-HSA-419812	Calcitonin-like ligand receptors	4.38E−03	*Calcrl;Adm*
R-HSA-73857	RNA polymerase II transcription	5.29E−03	*Mmp13;Nr3c1;Ccnd3;Prr5;Zfp385a;Zbtb16;Bdnf;Vdr;Fkbp5;Runx2;Tead4*
R-HSA-8939902	Regulation of RUNX2 expression and activity	6.51E−03	*Nr3c1;Runx2*
R-HSA-6783783	Interleukin-10 signaling	7.35E−03	*Ccl2;Ptgs2*
R-HSA-8986944	Transcriptional regulation by MECP2	1.22E−02	*Bdnf;Fkbp5*
R-HSA-1474290	Collagen formation	1.39E−02	*Mmp13;Mmp3;Col8a1*
R-HSA-74160	Gene expression (Transcription)	1.59E−02	*Mmp13;Nr3c1;Ccnd3;Prr5;Zfp385a;Zbtb16;Bdnf;Vdr;Fkbp5;Runx2;Tead4*
R-HSA-9006931	Signaling by nuclear receptors	2.28E−02	*Cav2;Mmp3;Pdk4;Dhrs9;Fkbp5*
R-HSA-5362517	Signaling by retinoic acid	2.64E−02	*Pdk4;Dhrs9*
R-HSA-9020591	Interleukin-12 signaling	3.88E−02	*Vamp7;Serpinb2*
R-HSA-4090294	SUMOylation of intracellular receptors	4.91E−02	*Nr3c1;Vdr*

### MCH signals to circadian clock genes in ciliated 3T3-L1 cells

**Figure 7:**
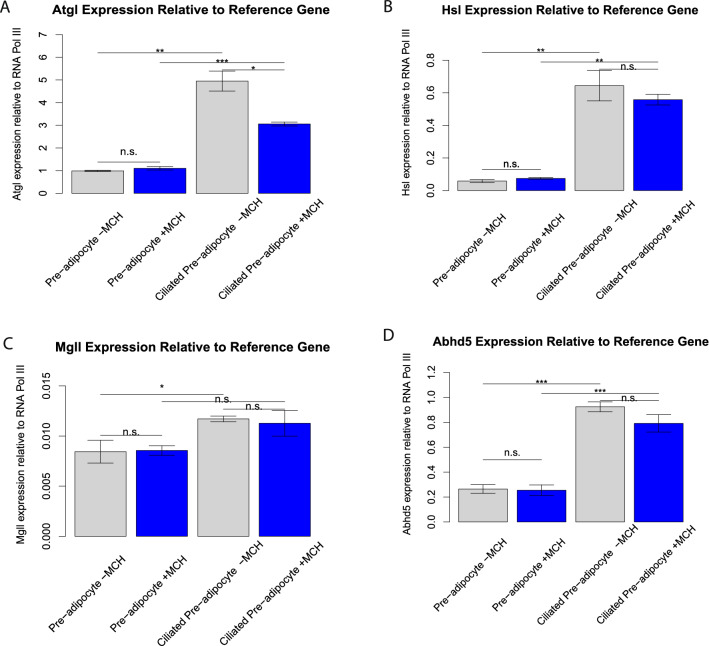
Expression of lipases compared to reference gene, analyzed by qPCR. (**A**) *atgl* expression is increased in differentiating, ciliated 3T3-L1 cells compared non-ciliated and MCH treatment significantly diminishes this upregulation (*p* < 0.01). (**B**) *hsl* expression follows the same induction pattern as *atgl*, but MCH treatment doesn’t have an effect. (**C**) Expression levels of *mgll* are more subtly increased with the appearance of primary cilia, with MCH treatment having an insignificant effect on expression at either timepoint. (**D**) Expression levels of the *atgl* co-factor *abhd5* increase with cilia formation and MCH significantly reduces this effect, similar to that observed with *atgl.* * *p* < 0.05; **p < 0.01; ***p < 0.001; n.s. not significant; n = 3.

**Figure 8:**
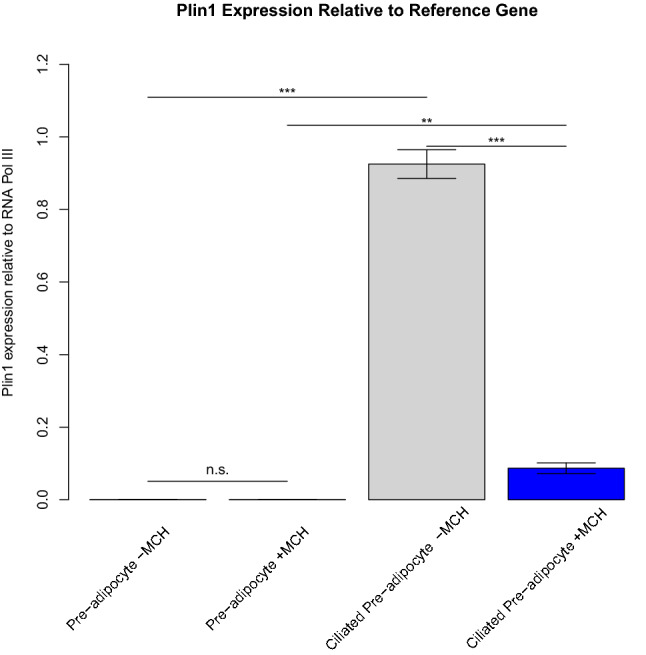
Expression of *plin1* normalized to reference gene, analyzed by qPCR. Gene expression is markedly increased in ciliated cells compared to non-ciliated cells regardless of MCH treatment. However, in ciliated cells, this increase in expression is significantly attenuated with 100 nM MCH treatment for 6 h. *p < 0.05; **p < 0.01; ***p < 0.001; n.s. not significant; n = 3.

Molecular circadian clocks establish rhythmic patterns in cells and tissues to achieve cyclic signaling. MCH signaling caused a small, but significant downregulation of the expression of all three of these genes only in ciliated pre-adipocytes, suggesting a potential connection between primary cilia and circadian clocks. According to Grimaldi et al., the circadian clock regulates key genes responsible for lipolysis^38^. In an effort to establish a connection between MCH signaling, clock gene inhibition, and lipolysis, we followed MCH-mediated gene expression changes of key lipases (*atgl, hsl, mgll* and *abhd5*) expressed in non-ciliated and ciliated 3T3-L1 cells by qPCR. As shown in Fig. 7, adipose triglyceride lipase (*atgl*) and the hydrolase that activates it (*abhd5*) are normally upregulated when adipogenesis is initiated. MCH signaling significantly downregulates that response. Perilipins are abundant proteins that serve to protect the lipid droplet from lipases. Perilipin 1 is expressed in both white and brown adipose tissue and acts to sequester ABHD5 from ATGL in unstimulated cells. Upregulation of *plin1*, the gene for perilipin 1, is significantly diminished by MCH treatment of ciliated pre-adipocytes as shown in Fig. 8. Together, these data present a model whereby activation of MCHR1on transient primary cilia of differentiating adipocytes dampens the adipogenic signal. Recently, a 24-h temporal gene expression analysis was completed to elucidate the role of circadian rhythms in human white adipose tissue. Despite the small sample size, rhythmicity in gene expression was quite evident in their study^39^. The propensity for MCH receptor 1 to generate signals in ciliated pre-adipocytes that influence circadian rhythm gene expression should be taken into consideration as clinical antagonists are explored for treatment of obesity and metabolic syndromes.

## Conclusion

Both ciliated and non-ciliated 3T3-L1 pre-adipocytes responded to MCH, however those responses were quite different, indicating that translocation of MCH receptor 1 to primary cilia during early adipogenesis significantly alters its signal transduction pathway, with the potential to influence adipose tissue development from primary cilia. We discovered that MCH signaling in ciliated pre-adipocytes inhibits gene expression of well-known drivers of adipogenesis and predict that the unique signaling environment of the primary cilium is what enables MCH receptors to participate in the adipogenic decision. This study reveals the importance of studying endogenous GPCRs in all cellular contexts involved in tissue development.

## Methods

### Tissue culture

Low passage mouse 3T3-L1 Pre-adipocytes (ATCC CL-173) were cultured as a tissue monolayer using high glucose DMEM^-^ media (ATCC) containing 10% bovine calf serum (Atlanta Biological) without antibiotics. Cells were fed every three days and passaged when they were subconfluent. Culture conditions were set at 37 °C, 5% CO_2_, and 80% humidity.

### Induction of 3T3-L1 adipogenesis

3T3-L1 pre-adipocytes were initially plated and grown to about 70% confluence in maintenance media. Then cells were transferred to DMEM (ATCC) + 10% fetal bovine serum (Atlanta Biological) with 10 μg/ml insulin (SantaCruz), 115 μg/ml isobutylmethylxanthine (Acros), and 400 ng/ml dexamethasone (AlfaAesar) and incubated for 2 days in a 37 °C, 5% CO_2_ incubator to induce primary cilia formation. For some experiments, induction media was withdrawn on Day 2 and replaced with DMEM + 10% fetal bovine serum with 250 ng/ml insulin for two days.

### Transfection

Cell transfections were performed on cells plated for at least 24 h with LipoD293 reagent following the recommended protocol from SignaGen. Media was changed 5 h post-transfection and experiments were run approximately 48 h post-transfection. The MCHR1-eYFP plasmid was a generous gift of Dr. Graeme Milligan (University of Glasgow).

### Confocal and fluorescence microscopy

Confocal Imaging was performed with the structured light imaging OptiGrid confocal microscope with Image ProPlus software. In some cases, fixed cell images were taken using a Zeiss Axiocam Mrm fluorescence microscope with AxioView imaging software.

### Total RNA extraction

Prior to collection of RNA, we verified that these low passage 3T3-L1 cells retained the capacity to differentiate. 3T3-L1 preadipocytes were grown on 60 mm tissue culture dishes. Total RNA was harvested on Days 0 and 2 of differentiation. Six hours prior to collection, cells were treated with or without 100 nM MCH in differentiation media. Cells were then harvested on ice, and a Qiagen EZ Shredder was used in conjunction with the RNeasy mini kit (Qiagen) per manufacturer’s instructions to homogenize the cells and collect the Total RNA. An on-column DNase digestion (Qiagen) was performed to minimize genomic DNA contamination. Each 60 mm dish of monolayer cells yielded 80–100 μl volume of 50 ng/μl total RNA in nuclease-free water. RNA samples were held at − 70 °C. To confirm the quality and quantity of RNA extracted by this procedure, all samples were analyzed on an Agilent 2100 Bioanalyzer at University of Rochester Functional Genomics Center, Rochester, NY. The RNA Integrity numbers (RINs) ranaged from 9.3 to 10.0 for 12 RNA samples [Figure S1]. Subsequent quantifications were performed on a Nanodrop2000 (Thermofisher).

### One-step reverse-transcriptase PCR

Reverse-transcriptase PCR was employed to test specificity of initial primers. Quanta Biosciences qScript XLT One-Step RT-PCR kit was used for per manufacturer’s instructions. Each 25 μl reaction received 12.5 µl of 2× Toughmix reagent, 1 µl reverse-transcriptase, 3 µl of each forward and reverse primers (5 µM each, IDT), 2.5 µl of nuclease-free water, and 3 µl of total RNA. Samples were loaded into a BioRad PCRexpress thermal cycler and run as follows: 20 min at 48 °C_,_ 3 min at 94 °C (1 cycle); 15 s at 94 °C, 45 s at 55–61 °C based on primer-specific annealing temperature, and 30–60 s at 70 °C depending on expected product length (34 cycles); 15 s at 94 °C, 45 s at 55–61 °C based on primer-specific annealing temperature, and 2 min at 70 °C (1 cycle). Agarose gel electrophoresis was used to confirm the presence of the desired PCR product, utilizing agarose concentrations of 1.5% and 0.5 µg/µl ethidium bromide.

### Quantitative PCR

In preparation for quantitative PCR, cDNA was generated from 3T3-L1 RNA stocks using qScript cDNA Supermix (Quanta Biosciences, Gaithersburg, MD). Each 20 µl reaction consisted of 4 µl cDNA Supermix, 4 µl RNA template, and 12 µl nuclease-free water. The reactions were placed in thermocycler for a single three-step cycle: a 5-min step at 25 °C, a 30-min step at 42 °C, and a 5 min step at 85 °C. Following the cycle, each cDNA sample was diluted in nuclease-free water and utilized for subsequent qPCR analysis. For storage, 5 µl of 10 µM Tris/0.1 µM EDTA buffer was added and samples were held at − 20 °C.

Early qPCR analyses were performed using PerfeCTa SYBR Green Supermix for iQ (Quanta Biosciences) chemistry using a BioRad MyiQ Thermocycler. Each 25 µl reaction consisted of 12.5 µl PerfeCTa SYBR Green Supermix (2×), 0.97 *μl* each forward and reverse primers (300 nM final concentration), 2 µl undiluted cDNA template, and 8.56 μl nuclease-free water. Quantitative PCR cycles consisted of a single denaturation cycle of 2.5 min at 95 °C, followed by 40 cycles of denaturation for 12 s at 95 °C and an annealing/extension step of 30 s at 59–62 °C. Melt curve analysis was performed following qPCR runs and ranged from 55 to 95 °C, increasing by 0.5 °C every 30 s step. C_T_ baseline was determined via automatic baseline cycle calculation and C_T_ values determined within the BioRad iQ5 software. Dynamic well factors were determined from each individual PCR plate. Gene expression data obtained from qPCR was used in conjunction with the −ΔΔC_T_ method described by others^40^. Student’s t-test was used for statistical analysis of qPCR data comparisons, n = 3 for each, unless otherwise noted.

Later qPCR analyses were performed with PowerUp SYBR Green Master Mix using the Applied Biosystems QuantStudio 3 (Thermo Fisher Scientific). Each 25 µl reaction consisted of 12.5 µl PowerUp SYBR Green Master Mix (2×), 1 μl each forward and reverse primers (400 nM final concentration), 2.5 µl cDNA template (optimal concentration for each primer set was determined using standard dilution series), and 800A0μl nuclease-free water and were prepared in quadruplicate following the same qPCR cycle design as previously described. Data was stored in the Thermo Fisher Connect Cloud and analyzed with the Relative Quantitation qPCR Application, where ΔΔC_T_ was calculated.

All primers were obtained from Integrated DNA Technologies. Upon receipt, lyophilized DNA primers were briefly centrifuged and resuspended in TE buffer (10 µM Tris, 1 µM EDTA) to a stock concentration of 100 µM. Standard curves were used to validate all qPCR primers and melt curve analyses were used to identify off-target PCR products. Primer sequences are available in Supplementary Table 1.

### RNA-Seq and bioinformatic analysis

Total RNA from twelve samples were sent to the Center for Genome Research and Biocomputing (CGRB) at Oregon State University for library construction and stranded-specific sequencing on an Illumina HiSeq3000 as previously reported^19^. Library construction was described in detail by Shen et al.^4^. Sequencing libraries were prepared with a WaferGen PrepXTM mRNA Strand-Specific Library Preparation Protocol (WaferGen Biosystem). As a result, all libraries are strand-specific and the direction of sequencing reads were identical the original direction of transcripts or mRNAs. All 12 libraries were barcoded and sequenced in one lane with 151 base-pair single-end.

Approximately 400 million 151-base reads were obtained for all 12 samples. Each sample had about 33 million reads in average after demultiplex ranging from 27 to 48 million [Supplementary Figure S2]. FastQC^41^ was used to check the quality of sequencing reads. Bases at the beginning and the tailing part of reads were trimmed since they had lower quality scores. The remaining bases of 7–106 were kept for each read containing 100 bases. Further analysis followed the procedures as described by others^4^. Briefly, all the reads were mapped to mouse mm10 assembly with RefSeq gene annotation using STAR^19^. HTSeq were employed to count the number of reads assigned to each gene^42^. After alignment, there were about 27.8 million in average mapped to the reference, which were corresponding to a mapping rate averaging 84%, varying from 78 to 88% [Supplementary Table 3]. The genes with very low number of reads or no reads were filtered out based on data distribution using density plotting (data not shown) to ensure the reliable data analysis. Transcript-specific variance estimates were obtained by fitting the negative binomial model implemented in DESeq2^43^ and significantly differentially expressed genes were determined with Wald statistics among the biological replicates. Those genes were functionally analyzed using DAVID (The Database for Annotation, Visualization, and Integrated Discovery)^19^, PANTHER^28^, and Reactome^29^. The averages of the data were reported ± the standard error of the mean (SEM). Student’s t-test was performed to determine statistical significance with data scoring equal to or greater than the 95th percentile considered significant.

## Supplementary Information

Supplementary Information

## Data Availability

The RNA-Seq expression data for Day 0 and Day 2 differentiating 3T3-L1 cell transcriptomes is available at GEO accession number GSE145452.
