# Supplementary material for: Transcriptome analysis of ciliary-dependent MCH signaling in differentiating 3T3-L1 pre-adipocytes
Source: Sci Rep. 2021 Mar 1;11:4880. doi: 10.1038/s41598-021-84138-4 (PMC7921120; doi:10.1038/s41598-021-84138-4)
Supplement: Supplementary file 1 — Supplementary Information [file 41598_2021_84138_MOESM1_ESM.docx]

**Transcriptome Analysis of Ciliary-Dependent**

**MCH Signaling in Differentiating 3T3-L1 Pre-adipocytes**

Laurie B. Cook*, Henry D. Ophardt, Rongkun Shen, Bryan H. Pratt, Stacy Wicks, Lucas A. Galbier,

**Appendix**

**Supplementary Figure S1**

**RNA quality from Bioanalyzer 2100 with RNA Integrity Numbers (RINs)**


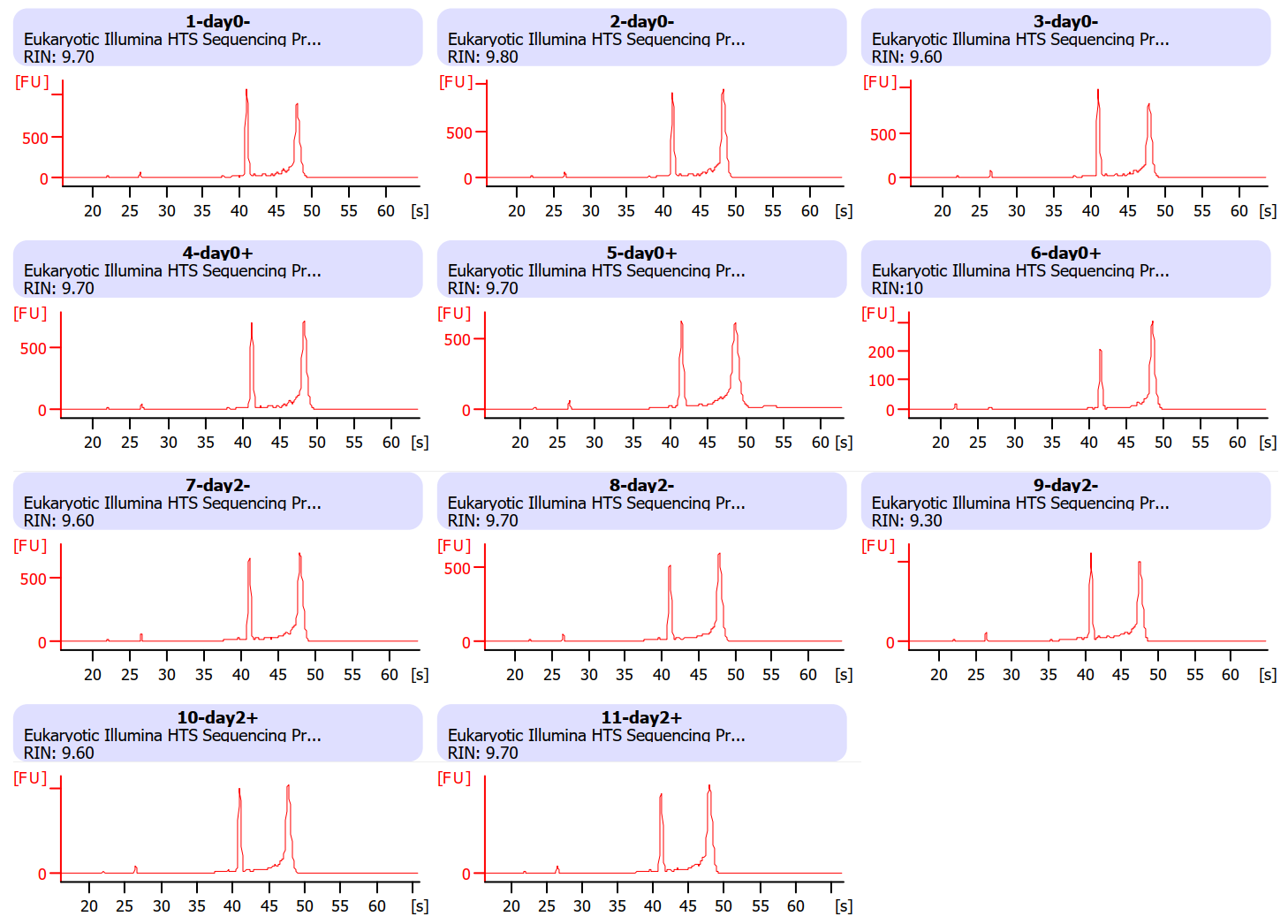


| Supplementary Table 1 - Reactome Pathway Analysis of Genes Significantly Changed by MCH in Non-Ciliated 3T3-L1 Cells | | | |
| --- | --- | --- | --- |
| Pathway Identifier | **Pathway Name** | **p-value** | **Genes Found in Pathway** |
| R-HSA-380320 | Recruitment of NuMA to mitotic centrosomes | 1.48E-03 | *Cep63; Tuba1c;Hsp90aa1* |
| R-HSA-168273 | Influenza Viral RNA Transcription and Replication | 1.90E-03 | *Rps14; Rps18;Rplp1;Polr2g;Hsp90aa1* |
| R-HSA-2408522 | Selenoamino acid metabolism | 2.20E-03 | *Rps14; Rps18;Rplp1;Eef1e1;Sephs2* |
| R-HSA-2408557 | Selenocysteine synthesis | 2.57E-03 | *Rps14;Rps18;Rplp1;Sephs2* |
| R-HSA-168255 | Influenza Life Cycle | 2.59E-03 | *Rps14; Rps18;Rplp1;Polr2g;Hsp90aa1* |
| R-HSA-1799339 | SRP-dependent cotranslational protein targeting to membrane | 3.09E-03 | *Rps14;Spcs1;Rps18;Rplp1* |
| R-HSA-168254 | Influenza Infection | 3.30E-03 | *Rps14; Rps18;Rplp1;Polr2g;Hsp90aa1* |
| R-HSA-5617833 | Cilium Assembly | 3.98E-03 | *Cep63;Tuba1c;Rab8a;Hsp90aa1* |
| R-HSA-69275 | G2/M Transition | 4.31E-03 | *Cep63;Tuba1c;Rab8a;Hsp90aa1* |
| R-HSA-453274 | Mitotic G2-G2/M phases | 4.48E-03 | *Cep63;Tuba1c;Rab8a;Hsp90aa1* |
| R-HSA-3371497 | HSP90 chaperone cycle for steroid hormone receptors (SHR) | 5.36E-03 | *Tuba1c;Hsp90aa1* |
| R-HSA-1445148 | Translocation of SLC2A4 (GLUT4) to the plasma membrane | 7.47E-03 | *Tuba1c;Rab8a* |
| R-HSA-69278 | Cell Cycle, Mitotic | 7.64E-03 | *Rfc4;Cep63;Top2a;Tuba1c;Rab8a;Hsp90aa1* |
| R-HSA-5637812 | Signaling by EGFRvIII in Cancer | 7.69E-03 | *Cdc37;Hsp90aa1* |
| R-HSA-5637810 | Constitutive Signaling by EGFRvIII | 7.69E-03 | *Cdc37;Hsp90aa1* |
| R-HSA-8852276 | The role of GTSE1 in G2/M progression after G2 checkpoint | 8.54E-03 | *Tuba1c;Hsp90aa1* |
| R-HSA-5601884 | PIWI-interacting RNA (piRNA) biogenesis | 1.08E-02 | *Polr2g;Hsp90aa1* |
| R-HSA-2565942 | Regulation of PLK1 Activity at G2/M Transition | 1.13E-02 | *Cep63;Rab8a;Hsp90aa1* |
| R-HSA-156902 | Peptide chain elongation | 1.30E-02 | *Rps14;Rps18;Rplp1* |
| R-HSA-5620912 | Anchoring of the basal body to the plasma membrane | 1.37E-02 | *Cep63;Rab8a;Hsp90aa1* |
| R-HSA-8863795 | Downregulation of ERBB2 signaling | 1.43E-02 | *Cdc37;Hsp90aa1* |
| R-HSA-975956 | Nonsense Mediated Decay (NMD) independent of the Exon Junction Complex (EJC) | 1.44E-02 | *Rps14;Rps18;Rplp1* |
| R-HSA-156842 | Eukaryotic Translation Elongation | 1.48E-02 | *Rps14;Rps18;Rplp1* |
| R-HSA-6791226 | Major pathway of rRNA processing in the nucleolus and cytosol | 1.52E-02 | *Rps14;Rps18;Rplp1;Snu13* |
| R-HSA-72689 | Formation of a pool of free 40S subunits | 1.64E-02 | *Rps14;Rps18;Rplp1* |
| R-HSA-72764 | Eukaryotic Translation Termination | 1.64E-02 | *Rps14;Rps18;Rplp1* |
| R-HSA-5637815 | Signaling by Ligand-Responsive EGFR Variants in Cancer | 1.66E-02 | *Cdc37;Hsp90aa1* |
| R-HSA-1236382 | Constitutive Signaling by Ligand-Responsive EGFR Cancer Variants | 1.66E-02 | *Cdc37;Hsp90aa1* |
| R-HSA-192823 | Viral mRNA Translation | 1.98E-02 | *Rps14;Rps18;Rplp1* |
| R-HSA-8868773 | rRNA processing in the nucleus and cytosol | 2.04E-02 | *Rps14;Rps18;Rplp1;Snu13* |
| R-HSA-68877 | Mitotic Prometaphase | 2.04E-02 | *Cep63;Tuba1c;Hsp90aa1* |
| R-HSA-1640170 | Cell Cycle | 2.06E-02 | *Rfc4;Cep63;Top2a;Tuba1c;Rab8a;Hsp90aa1* |
| R-HSA-1643713 | Signaling by EGFR in Cancer | 2.26E-02 | *Cdc37;Hsp90aa1* |
| R-HSA-72706 | GTP hydrolysis and joining of the 60S ribosomal subunit | 2.26E-02 | *Rps14;Rps18;Rplp1* |
| R-HSA-156827 | L13a-mediated translational silencing of Ceruloplasmin expression | 2.26E-02 | *Rps14;Rps18;Rplp1* |
| R-HSA-927802 | Nonsense-Mediated Decay (NMD) | 2.46E-02 | *Rps14;Rps18;Rplp1* |
| R-HSA-975957 | Nonsense Mediated Decay (NMD) enhanced by the Exon Junction Complex (EJC) | 2.46E-02 | *Rps14;Rps18;Rplp1* |
| R-HSA-5610787 | Hedgehog 'off' state | 2.46E-02 | *Itch;Tuba1c* |
| R-HSA-1852241 | Organelle biogenesis and maintenance | 2.66E-02 | *Cep63;Tuba1c;Rab8a;Hsp90aa1* |
| R-HSA-72766 | Translation | 2.78E-02 | *Rps14;Spcs1;Rps18;Rplp1;Eef1e1* |
| R-HSA-72613 | Eukaryotic Translation Initiation | 2.78E-02 | *Rps14;Rps18;Rplp1* |
| R-HSA-72737 | Cap-dependent Translation Initiation | 2.78E-02 | *Rps14;Rps18;Rplp1* |
| R-HSA-72695 | Formation of the ternary complex, and subsequently, the 43S complex | 3.03E-02 | *Rps14;Rps18* |
| R-HSA-72165 | mRNA Splicing - Minor Pathway | 3.24E-02 | *Polr2g;Snu13* |
| R-HSA-6799198 | Complex I biogenesis | 3.35E-02 | *Ndufb2;Ndufa2* |
| R-HSA-72312 | rRNA processing | 3.48E-02 | *Rps14;Rps18;Rplp1;Snu13* |
| R-HSA-72649 | Translation initiation complex formation | 3.90E-02 | *Rps14;Rps18* |
| R-HSA-72702 | Ribosomal scanning and start codon recognition | 4.13E-02 | *Rps14;Rps18* |
| R-HSA-6782210 | Gap-filling DNA repair synthesis and ligation in TC-NER | 4.36E-02 | *Rfc4;Polr2g* |
| R-HSA-72662 | Activation of the mRNA upon binding of the cap-binding complex and eIFs, and subsequent binding to 43S | 4.36E-02 | *Rps14;Rps18* |
| R-HSA-1227986 | Signaling by ERBB2 | 4.48E-02 | *Cdc37;Hsp90aa1* |
| R-HSA-6782135 | Dual incision in TC-NER | 4.60E-02 | *Rfc4;Polr2g* |
| R-HSA-380284 | Loss of proteins required for interphase microtubule organization from the centrosome | 4.97E-02 | *Cep63;Hsp90aa1* |
| R-HSA-380259 | Loss of Nlp from mitotic centrosomes | 4.97E-02 | *Cep63;Hsp90aa1* |
| R-HSA-6790901 | rRNA modification in the nucleus and cytosol | 4.97E-02 | *Rps14;Snu13* |

| Supplementary Table 2  qPCR Primer Sequences | | |
| --- | --- | --- |
| Gene | **Primer** | **Sequence (5' to 3')** |
| *atgl* | Forward | ttcgcaatctctaccgcctc |
|  | Reverse | gcaaagggttgggttggttc |
| *hsl* | Forward | aggatcgaaccgcagtc |
|  | Reverse | gtcttctgcgagtgtcacca |
| *mgll* | Forward | ctcggaacaagtcggaggtt |
|  | Reverse | cattgcctccactcttg |
| *abhd5* | Forward | cctcagtgcaggacctt |
|  | Reverse | ctgtctcaccacttggggtt |
| *plin1* | Forward | tgccttacctagctgctttctc |
|  | Reverse | ggtctccatccagcagggtt |
| *ppar−γ* | Forward | tctcctgttgacccagagca |
|  | Reverse | atcacggagaggtccacaga |
| *c/ebp-α* | Forward | ttcatggagaatgggggcac |
|  | Reverse | tagagctgcacactgccatt |
| *c/ebp-β* | Forward | gaagacggtggacaagctga |
|  | Reverse | ccacgtttgatccggattgc |
| *c/ebp-δ* | Forward | caccagggaaaagcaagcac |
|  | Reverse | gtccacaagcctgcactttg |
| *RNApol II* | Forward | agctgtggatcgaggctttt |
|  | Reverse | ccggagctatcaaaccgtca |
| *lcn2* | Forward | ctctgggcctcaaggacgacaac |
|  | Reverse | agccacactcaccacccattcag |
| *igfbp4* | Forward | ccattccaaactgtgaccgcaac |
|  | Reverse | ccgatccacacaccagcacttg |
| *gas7* | Forward | gccgctatgcctcagtggagaag |
|  | Reverse | tgtcctcctcggtcttgttgctc |

**Supplementary Figure S2**

**Density plot of read counts before and after filtering off the genes with very low read or none.**


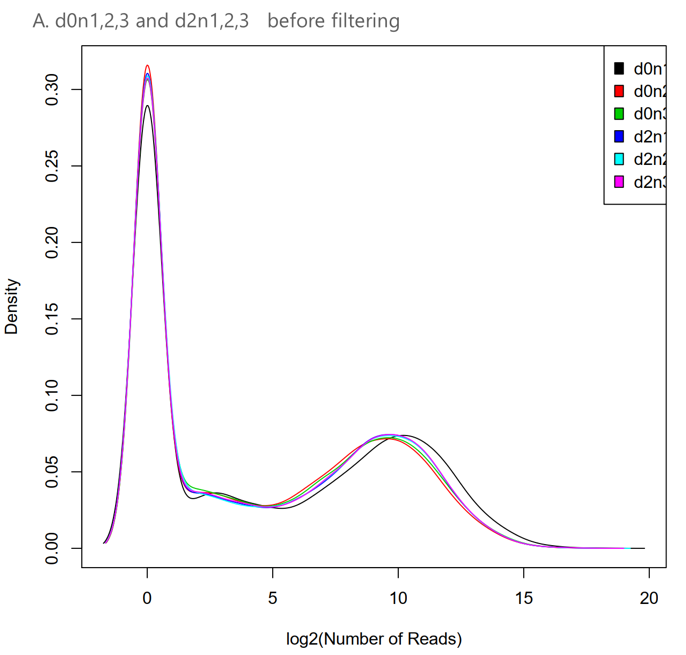

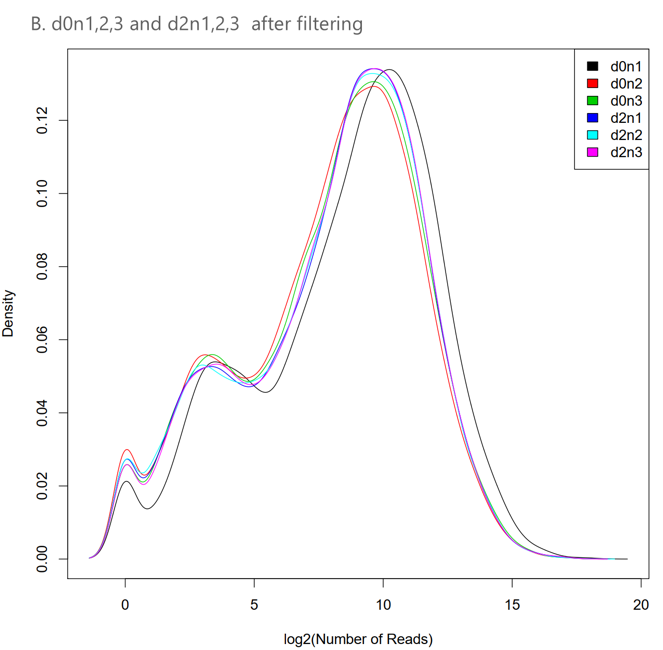


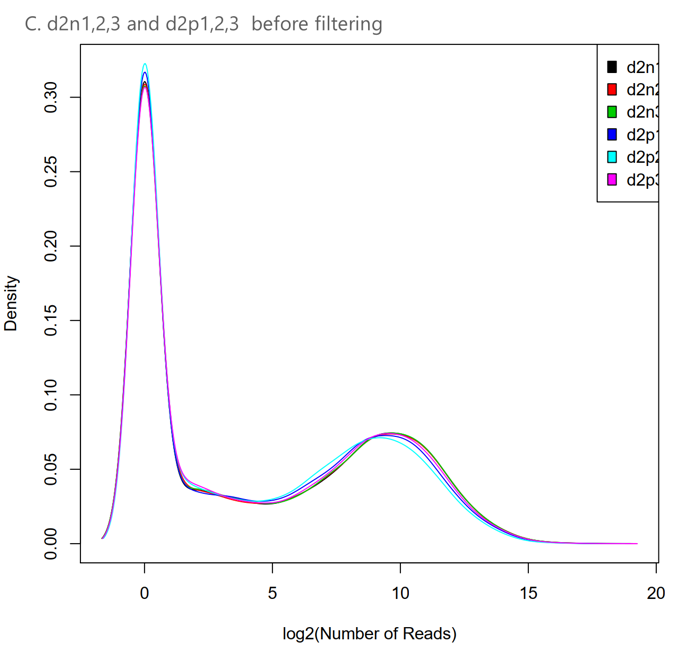

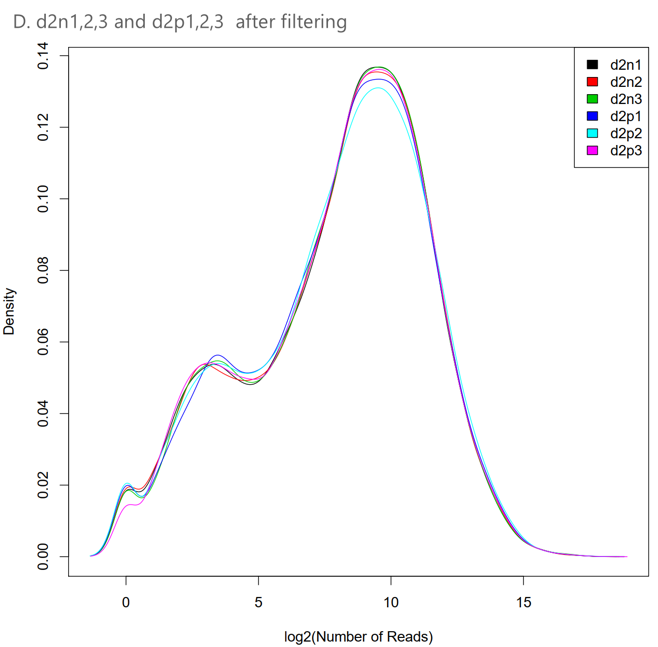


**Supplementary Table 3**

**The number of raw reads, mapped reads (in millions)**

**and mapping rates**

|  | d0n1 | d0n2 | d0n3 | d0p1 | d0p2 | d0p3 | d2n1 | d2n2 | d2n3 | d2p1 | d2p2 | d2p3 | Ave |
| --- | --- | --- | --- | --- | --- | --- | --- | --- | --- | --- | --- | --- | --- |
| Total mapped reads | 42.4 | 27.0 | 28.5 | 25.3 | 28.5 | 22.0 | 28.1 | 28.2 | 28.9 | 25.8 | 22.8 | 26.9 | **27.9** |
| Total raw reads | 48.7 | 31.3 | 33.4 | 30.9 | 34.2 | 27.0 | 31.9 | 33.4 | 33.0 | 30.3 | 29.1 | 31.7 | **32.9** |
| Mapping rate | 87.2% | 86.2% | 85.3% | 82.0% | 83.3% | 81.4% | 87.9% | 84.4% | 87.4% | 85.0% | 78.3% | 84.8% | **84.4%** |
